# The Acute Effects of Interval-Type Exercise on Glycemic Control in Type 2 Diabetes Subjects: Importance of Interval Length. A Controlled, Counterbalanced, Crossover Study

**DOI:** 10.1371/journal.pone.0163562

**Published:** 2016-10-03

**Authors:** Ida Jakobsen, Thomas P. J. Solomon, Kristian Karstoft

**Affiliations:** 1 The Centre of Inflammation and Metabolism and the Centre for Physical Activity Research, Rigshospitalet, University of Copenhagen, Copenhagen, Denmark; 2 School of Sport, Exercise, and Rehabilitation Sciences, University of Birmingham, Birmingham, United Kingdom; 3 Institute of Metabolism and Systems Research (IMSR), University of Birmingham, Birmingham, United Kingdom; Weill Cornell Medical College in Qatar, QATAR

## Abstract

Interval-type exercise is effective for improving glycemic control, but the optimal approach is unknown. The purpose of this study was to determine the importance of the interval length on changes in postprandial glycemic control following a single exercise bout. Twelve subjects with type 2 diabetes completed a cross-over study with three 1-hour interventions performed in a non-randomized but counter-balanced order: 1) Interval walking consisting of repeated cycles of 3 min slow (aiming for 54% of Peak oxygen consumption rate [VO_2_peak]) and 3 min fast (aiming for 89% of VO_2_peak) walking (IW3); 2) Interval walking consisting of repeated cycles of 1 min slow and 1 min fast walking (IW1) and 3) No walking (CON). The exercise interventions were matched with regards to walking speed, and VO_2_ and heart rate was assessed throughout all interventions. A 4-hour liquid mixed meal tolerance test commenced 30 min after each intervention, with blood samples taken regularly. IW3 and IW1 resulted in comparable mean VO_2_ and heart rates. Overall mean postprandial blood glucose levels were lower after IW3 compared to CON (10.3±3.0 vs. 11.1±3.3 mmol/L; P < 0.05), with no significant differences between IW1 (10.5±2.8 mmol/L) and CON or IW3 and IW1 (P > 0.05 for both). Conversely blood glucose levels at specific time points during the MMTT differed significantly following both IW3 and IW1 as compared to CON. Our findings support the previously found blood glucose lowering effect of IW3 and suggest that reducing the interval length, while keeping the walking speed and time spend on fast and slow walking constant, does not result in additional improvements.

**Trial Registration:**

ClinicalTrials.gov NCT02257190

## Introduction

Physical activity is part of the first line treatment in type 2 diabetes and the effect of physical activity on glycemic control is extensively investigated with well-documented beneficial effects [1,2]. The optimal training approach regarding type, duration and training intensities, however, is still not fully elucidated.

Subjects with type 2 diabetes are recommended to do moderate-intensity aerobic exercise at least three days per week with no more than two consecutive days without exercise [3]. This means that subjects with type 2 diabetes, who follow the recommendations, will for a substantial part of their life be acutely influenced by the last exercise bout. Therefore, the acute effects of exercise are important to investigate.

We have tested aerobic interval walking (IW) as a novel type of exercise and found that both a long-term exercise intervention [4,5] and a single exercise bout [6] is superior to energy-expenditure and time-duration matched continuous walking (CW) exercise for improving glycemic control in subjects with type 2 diabetes. IW, as we have tested it so far, consists of cycles of 3 min of slow and 3 min of fast walking (IW3). Our results are supported by other studies of interval-type interventions showing beneficial changes in metabolic variables when compared to continuous-type interventions [7,8].

The reason why IW is superior to CW upon improving glycemic control is unclear. In that context, at least two factors separate IW from CW: Peak exercise intensity and the alternating intensity pattern (the shift from low to high intensity and vice versa). Since some studies have found that continuous exercise with higher intensity results in greater improvements in metabolic variables compared to continuous exercise with lower intensity [9–12], the peak exercise intensity in IW may be responsible for the larger improvements in IW compared to CW. Conversely, other studies have found no differences between higher vs. lower continuous intensity exercise programs on metabolic variables [13–15], and one study has even found that a single lower intensity exercise session improves glycemic control more than a single exercise session with higher intensity [16]. Thus, with these inconsistencies of exercise intensity in mind, it may be speculated that the alternating intensity pattern (the length and the number of intervals) rather than the peak intensity is the responsible factor for the beneficial effects seen of interval-based exercise.

In that context, one study including overweight/obese men compared two high intensity interval training programs consisting of cycles of 1 min and 2 min duration, respectively [17]. Both programs showed improvements in insulin sensitivity compared to control, but there was no difference between the two programs. In this study however, the duration of each exercise session was of only 10 min and the subjects included were not diagnosed with type 2 diabetes. Another study found that longer intervals resulted in increased physiological strain and higher carbohydrate utilization [18]. This study, however, was performed in healthy subjects, and did not assess glycemic control in the period following exercise. As far as we are aware, no studies have examined glycemic control in subjects with type 2 diabetes after exercise sessions of different interval length but with the same time duration and intensity of the exercise.

Therefore, the aim of this study was to investigate whether the benefit from an acute interval exercise session on postprandial glycemic control can be increased if more intervals are performed. For this purpose we compared IW3 to an interval session consisting of repeated cycles of 1 min of slow and 1 min of fast walking (IW1) in subjects with type 2 diabetes. The two exercise sessions were matched with regards to walking speed and time spend on fast and slow walking. We hypothesized that both exercise sessions were superior to no walking upon improving glycemic control, and furthermore that IW1 might improve glycemic control more than IW3.

## Materials and Methods

### Subjects

Subjects with type 2 diabetes [3], age > 30 years and body mass index (BMI) between 18 and 40 kg/m^2^, were recruited to the study via advertisements. Exclusion criteria were the use of exogenous insulin, pregnancy, smoking, evidence of liver, renal, cardiopulmonary or thyroid disease, pre-menopausal status (for women) and contraindications to increased levels of physical activity [19].

All subjects underwent a medical screening including a medical examination, a blood chemistry screen, an oral glucose tolerance test (OGTT), a Dual x-ray absorptiometry (DXA) scan (Lunar Prodigy Advance, GE Healthcare, Madison, WI, USA) and a graded walking VO_2_peak-test with a portable indirect calorimetry system (Cosmed K4b^2^, Rome, Italy). Furthermore, subjects completed a physical activity questionnaire [20].

The VO_2_peak test consisted of three successive 3-min stages where subjects walked on flat ground with subjective velocities: slow, moderate and as fast as possible. Subjects were paced by an instructor to ensure that they walked as fast as possible during the last stage. The VO_2_peak was calculated as the mean oxygen consumption during the last min of the last stage [6,21].

Oral and written informed consent was obtained from all subjects. The study was approved by the ethical committee of the Capital Region of Denmark (H-1-2014-060) and prospectively registered at www.ClinicalTrials.gov (NCT02257190).

The sample size was based on our previous study evaluating differences in postprandial glycemic control after IW3 vs. CON [6]. In that study, an effect size of 1.10 mmol/l in mean glucose levels during the MMTT was found between IW3 and CON (Mean±standard deviation = 8.7±2.2 mmol/l and 9.9±2.7 mmol/l for IW and CON, respectively. Correlation between interventions = 0.92). To adjust for multiple comparisons, α was set at 0.017 (0.05/3). With a selected power (1-β) of 0.80, analysis (G*Power, v3.1.9.2, Düsseldorf, Germany) indicated that 12 subjects should complete the study.

### Trials

All subjects performed three trials in a non-randomized but counterbalanced order. Trials were identical except for the following interventions: 1) One hour of interval walking consisting of repeated cycles of 3 min slow and 3 min fast walking (IW3); 2) One hour of interval walking consisting of repeated cycles of 1 min slow and 1 min fast walking (IW1); and 3) No walking (CON). Neither the subjects, nor the study investigators were blinded. Trials were separated by at least one week. Subjects were asked to withhold anti-diabetic medication and to abstain from vigorous activity from two days before until the end of each trial day. Moreover, subjects were instructed to eat the same food and to refrain from caffeine and alcohol for 24 hours prior to each trial day. As such, all consumed food and beverages was measured and registered by the subjects in a 24 h diet record in order for them to replicate it exactly. In the beginning of each intervention day, the diet records were checked by the investigators and the subjects confirmed that no vigorous physical activity had been performed for the preceding 48 hours.

### Intervention day

Following an overnight fast (≥8 hours), subjects arrived at the laboratory, body weight was measured and an antecubital venous catheter for blood sampling was placed. Baseline blood samples were taken, and the intervention was initiated. During the IW1 and IW3 interventions, subjects walked on a treadmill with 1% incline, and during the control intervention subjects sat on a chair for one hour instead of walking. Breath-by-breath indirect calorimetry (Cosmed Quark, Rome, Italy) and heart rate monitoring (Cosmed, Wireless HR monitor) was carried out continuously during the interventions. The two exercise interventions were matched regarding time duration and walking speed.

In the end of both slow (t = 27 and 57 min) and fast (t = 30 and 60 min) intervals, subjects were asked to assess the current rate of perceived exertion (RPE) using a Borg scale [22]. Following the intervention subjects were additionally asked to rate the overall perceived exertion for the total intervention. After completing both exercise interventions subjects were asked which intervention they found was the harder one and which intervention they preferred.

After the intervention, subjects rested in a bed, and 30 min after termination of the intervention, a 4-hour standardized liquid mixed meal tolerance test (MMTT) was initiated (Nestlé Resource Komplett Näring 1.5, Frankfurt, Germany, 300 ml, 450 kcal; macronutrient composition: carbohydrates 55E%, protein 15E% and fat 30E%).

### Blood Sampling and Analysis

Blood samples were collected at baseline (t = 0), during both slow (t = 27 and 57 min) and fast (t = 30 and 60 min) intervals of the intervention and every 15^th^ minute throughout the MMTT. Glucose and lactate levels were analyzed immediately in heparinized blood (ABL800 FLEX analyser, Radiometer, Broenshoej, Denmark). Blood samples for subsequent analyses of insulin (Lithium Heparin tubes) were immediately placed on ice, centrifuged (2000 x *g*, at 4°C for 15 min), and plasma was extracted and stored at –80°C until analysis. Insulin levels were measured by electrochemiluminescense immunoassay (Cobas 8000, e602 modular, Roche, Basel, Switzerland).

### Trial order

**Fig 1:**
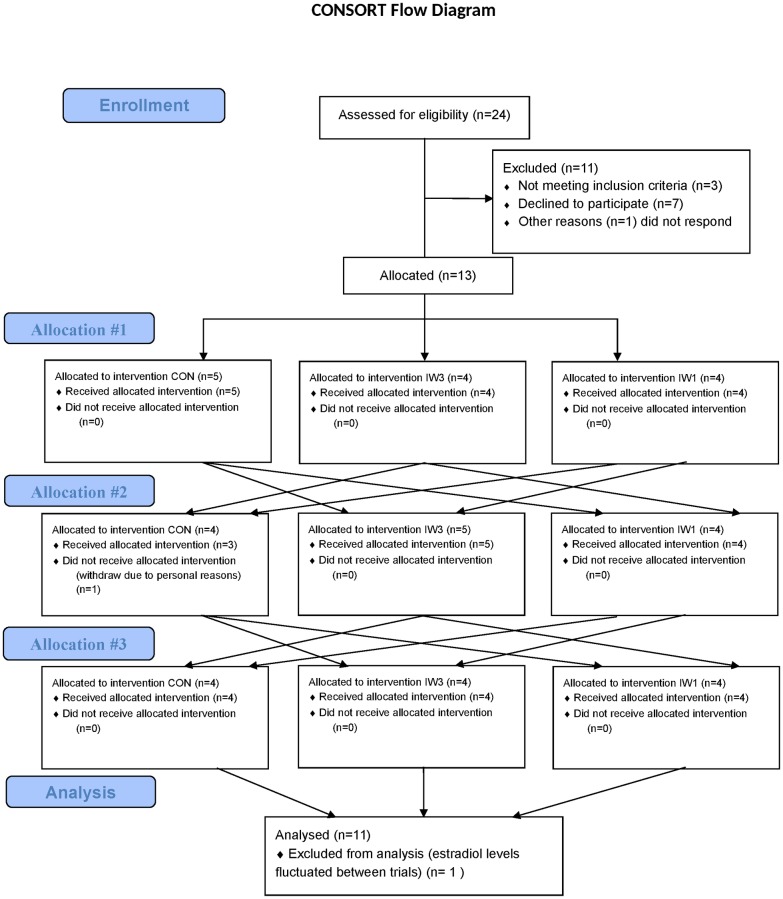
CONSORT flow diagram.

Concerning exercise intensity, the aim was to reach oxygen consumption rates at 54 and 89% of the individual VO_2_peak during the last minute of IW3 for slow and fast intervals, respectively. These intensities have previously shown efficient for improving postprandial glycemic control after an acute bout of IW3 in subjects with type 2 diabetes [5,6]. To ensure that the individual walking speeds corresponded to the correct oxygen consumption rates during IW3, the first 3 subjects started with the IW3 intervention and the walking speed was adjusted continuously to reach correct intensities throughout the exercise bout, while the walking speeds were registered. The walking speeds found during IW3 for these 3 subjects were then replicated during the following IW1 exercise intervention. Oxygen consumption rates relative to VO_2_peak during fast and slow IW1 intervals in these 3 subjects were subsequently used to determine oxygen consumption rates during fast and slow IW1 intervals, for upcoming IW1 exercise bouts in subjects where IW1 was performed before IW3. The remaining 10 subjects were allocated to trials in a way by which the entire study was counterbalanced (see Fig 1).

### Study outcomes

The primary outcome was postprandial glycemic control following each intervention.

Secondary outcomes included insulin levels and insulin sensitivity measures after each intervention, and ancillary analyses included RPE and preferred exercise intervention.

### Calculations

Total oxygen consumption and HR were calculated as mean during the entire intervention. For IW1 and IW3 oxygen consumption were also calculated as mean during both the slow and the fast intervals. Oxygen consumption and HR were furthermore calculated during the last minute of both the slow and the fast intervals for IW3, and compared to the corresponding minutes during IW1.

Glucose, insulin and lactate values at baseline and during exercise are reported as absolute values. After the interventions and during the MMTT, glucose values are reported as absolute as well as incremental values (baseline subtracted).

Insulin sensitivity was estimated using different insulin sensitivity indices before/during the MMTT [23–26].

### Statistics

Variables primarily relevant to the exercise trials (VO2, HR, lactate and RPE) were compared using Student’s paired t-test.

Variables relevant to all trials were compared using one-way (trial as factor) repeated-measures analysis of variance (RM-ANOVA), with Bonferroni-corrected post hoc tests applied to identify significant differences between interventions.

All statistical analyses were performed using Prism v6 (Graphpad, San Diego, CA).

Results are reported as mean±SD. A P-value < 0.05 (two-tailed) was considered significant.

## Results

### Subjects

Recruitment started at October 1^st^, 2014, and last intervention day was May 31^st^, 2015. Thirteen subjects were recruited to the study. One subject dropped out due to personal reasons. The remaining twelve subjects completed all three trial days as planned. For one subject, the estradiol levels fluctuated substantially between intervention days which raised doubt about her menopausal status. Since the female menstrual cycle may influence glycemic control [27,28], this subject was excluded from the data analysis. As such, eleven subjects were included in the data analysis and their baseline characteristics are shown in Table 1.

**Table 1:** Baseline characteristics.

N	11
Sex (M/F)	6/5
Age (y)	61.6 ± 8.3
Time since diagnosis (y)	7.0 ± 3.7
MLTPAQ (kcal/day)	265.5 ± 154.7
Body composition	
Body mass (kg)	87.1 ± 20.4
BMI (kg/m^2^)	29.0 ± 5.0
Lean body mass (kg)	56.1 ± 13.0
Body fat content (%)	35.8 ± 9.9
Medication	
No diabetes medication	2
Metformin	8
Sulfonylureas	2
DPP4 inhibitors	4
Glycemic control	
Fasting glucose (mmol/L)	7.2 ± 1.1
Fasting insulin (pmol/L)	109.2 ± 58.1
2 h OGTT glucose (mmol/L)	14.7 ± 4.3
HbA1c (mmol/mol)	46.5 ± 6.3
Fitness variables	
VO_2_peak (L O_2_/min)	2.0 ± 0.5

Data are mean ± SD.

MLTPAQ = Minnesota Leisure Time Physical activity [20], BMI = Body Mass Index, OGTT = oral glucose tolerance test, HbA1c = Hemoglobin A1c, VO_2_peak = peak oxygen consumption rate during walking on flat ground.

None of the subjects had changes in their medication throughout the study, and no adverse events were seen during the study.

### Interventions

Exercise characteristics are shown in Table 2.

**Table 2:** Exercise characteristics.

		IW1	IW3
Speed (km/h)	Slow	3.7 ± 0.7	3.7 ± 0.7
	Fast	6.1 ± 0.7	6.1 ± 0.7
	Mean	4.9 ± 0.7	4.9 ± 0.7
VO_2_ (ml/min)	Slow	1412 ± 380	1248 ± 335 #
	Fast	1417 ± 375	1550 ± 444 #
	Mean	1415 ± 376	1399 ± 389
VO_2_ (ml/min)*	Slow	1435 ± 382	1101 ± 290 #
	Fast	1416 ± 378	1694 ± 486 #
RER	Slow	0.81 ± 0.04	0.84 ± 0.03 #
	Fast	0.84 ± 0.04	0.82 ± 0.03
	Mean	0.82 ± 0.04	0.83 ± 0.03
HR (bpm)	Slow	104.8 ± 11.3	101.7 ± 10.2
	Fast	104.2 ± 10.7	111.6 ± 10.4 #
	Mean	104.5 ± 11.0	106.6 ± 10.1
Lactate (mmol/L)	Slow	1.36 ± 0.30	1.58 ± 0.43 #
	Fast	1.30 ± 0.32	1.58 ± 0.44 #
	Mean	1.33 ± 0.31	1.58 ± 0.43 #
RPE (a.u.)	Slow	11.1 ± 1.5	11.2 ± 1.5
	Fast	13.3 ± 1.5	13.5 ± 1.8
	Mean	12.2 ± 1.4	12.3 ± 1.4
	Total	12.9 ± 1.9	13.0 ± 1.3

^#^ P < 0.05; IW1 vs. IW3

*Oxygen consumption rate during the slow and fast intervals of the IW3 intervention measured during the last minute in each of the 3 minute intervals. For IW1, oxygen consumption rate during the corresponding minutes was calculated.

Data are mean ± SD.

VO_2_ = oxygen consumption rate, RER = respiratory exchange ratio, HR = heart rate, bpm = beats per minute, RPE = Rate of Perceived Exertion.

**Fig 2:**
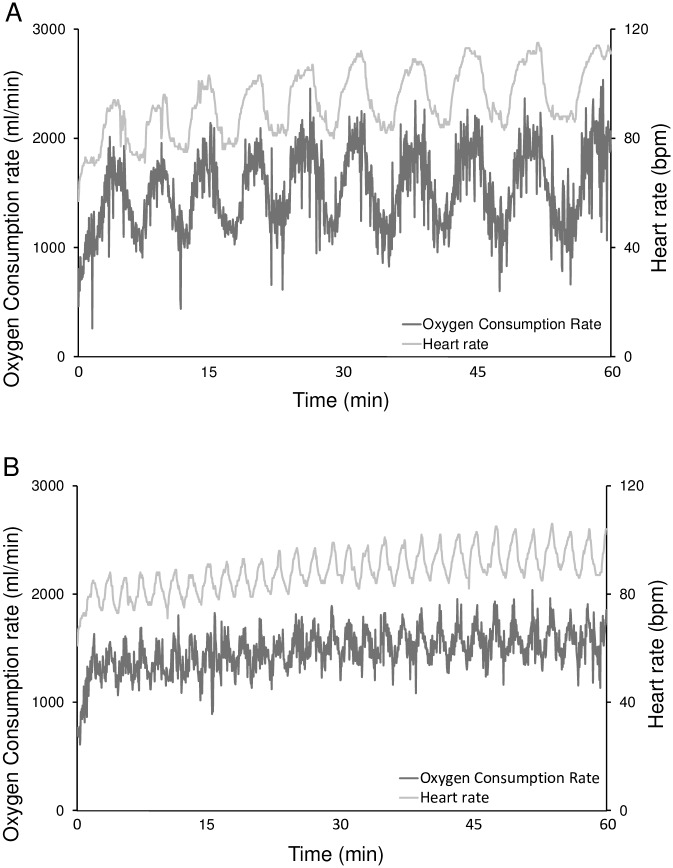
Representative examples of IW3 (A) and IW1 (B) from the same subject. Subjects with type 2 diabetes completed a cross-over study where they underwent three 1-hour interventions in a counterbalanced order: 1) Interval walking consisting of repeated cycles of 3 min slow and 3 min fast walking (IW3); 2) Interval walking consisting of repeated cycles of 1 min slow and 1 min fast walking (IW1) and 3) No walking (CON). Oxygen consumption and heart rate were measured throughout the interventions.

Mean VO_2_ during exercise was not different between IW1 and IW3, whereas VO_2_ was lower and higher during slow and fast intervals respectively, when comparing IW3 to IW1 (Fig 2 and Table 2). This was even more pronounced when the last minute of the IW3 intervals (during which VO_2_ reached 54.5±7.4 and 83.0±12.5% of the VO_2_peak during slow and fast intervals respectively), was compared to the corresponding minutes of IW1 (where 70.6±8.3 and 69.8±8.5% of VO_2_peak were reached for slow and fast intervals respectively).

There was no significant difference in body weight between trials prior to the interventions (87.0±20.1 kg vs. 86.9±20.6 kg vs. 87.2±20.6 kg for CON, IW1 and IW3, respectively; P > 0.05 for all).

During the exercise interventions, VO_2_ was at all times higher than during the control intervention (data not shown).

Mean HR during exercise was not significantly different between IW1 and IW3 (P > 0.05).

IW3 resulted in higher HR during the fast intervals as compared to IW1 (P < 0.01), whereas IW3 tended to be lower compared to IW1 during the slow intervals (P < 0.1). When comparing the last minute of the IW3 intervals to the corresponding minutes of IW1 there was a significant difference for both the slow (P < 0.01) and for the fast (P < 0.001) intervals.

Throughout the exercise interventions, HR was at all times higher than during the control intervention (66.5 bpm±9.4, P<0.01 for both).

Blood lactate levels during the exercise intervention were at all times higher in IW3 compared to IW1, and for both IW3 and IW1 at all times higher compared to control (0.96±0.06, P<0.01 for both).

### Glycemic control

**Fig 3:**
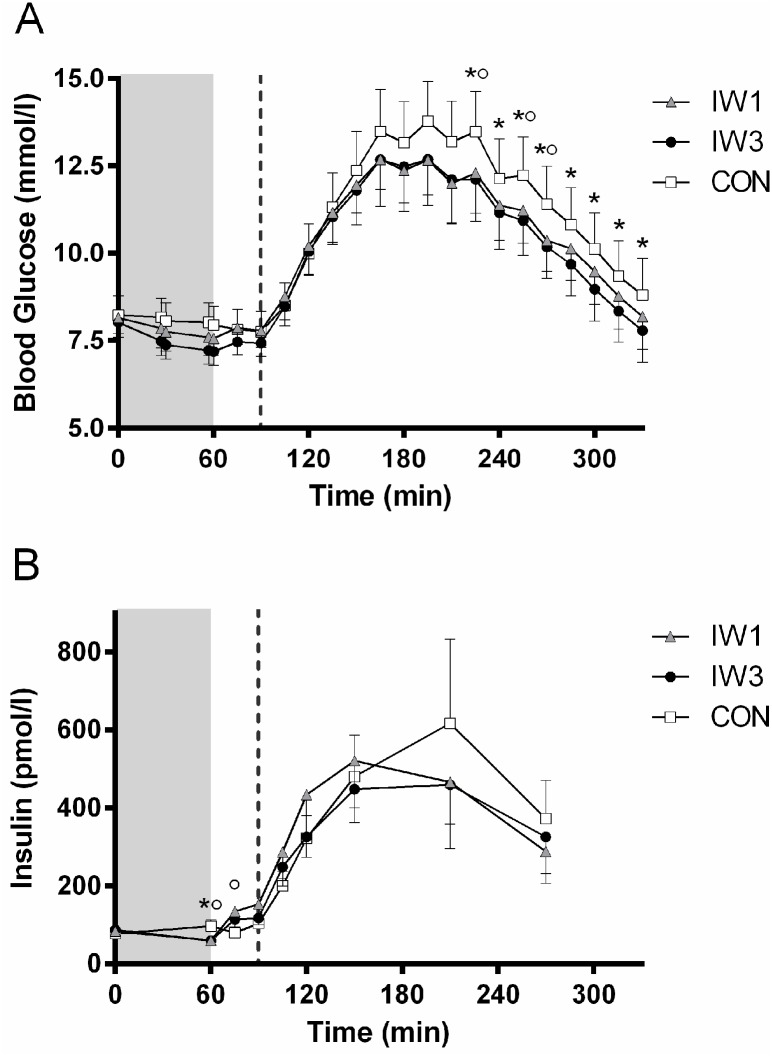
Blood glucose (A) and plasma insulin (B) levels. Subjects with type 2 diabetes completed a cross-over study where they underwent three 1-hour interventions in a counterbalanced order: 1) Interval walking consisting of repeated cycles of 3 min slow and 3 min fast walking (IW3); 2) Interval walking consisting of repeated cycles of 1 min slow and 1 min fast walking (IW1) and 3) No walking (CON). A liquid mixed meal tolerance test (MMTT) was started 30 minutes after end of the intervention. The shaded area indicates the intervention (t = 0–60 min), whereas the dotted vertical line indicates start of the MMTT (t = 90 min). Data are presented as mean ± SEM. Differences at specific time points (P<0.05) were analysed by one-way repeated measures ANOVA with Bonferroni-corrected post hoc tests. * indicates CON vs. IW3 and ° indicates CON vs. IW1.

There were no significant differences in baseline blood glucose levels between trial days before the interventions (8.2±1.8 mmol/L vs. 8.2±1.5 mmol/L vs. 8.0±1.4 mmol/L for CON, IW1 and IW3, respectively; P > 0.05 for all) (Fig 3A).

**Fig 4:**
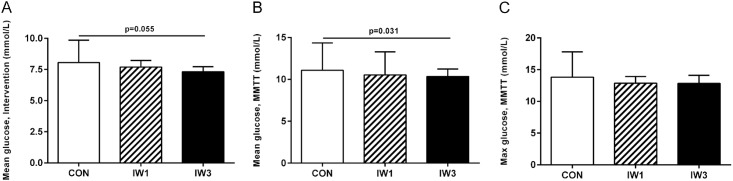
Mean blood glucose levels during the intervention (A) and the MMTT (B) and maximal blood glucose levels during the MMTT (C). Subjects with type 2 diabetes completed a cross-over study where they underwent three 1-hour interventions in a counterbalanced order: 1) Interval walking consisting of repeated cycles of 3 min slow and 3 min fast walking (IW3); 2) Interval walking consisting of repeated cycles of 1 min slow and 1 min fast walking (IW1) and 3) Control (no walking; CON). A 4 hour mixed meal tolerance test (MMTT) was started 30 minutes after end of the intervention (t = 90 min). Data are presented as mean ± SD. Differences were analysed by one-way repeated measures ANOVA with Bonferroni-corrected post hoc tests.

For statistical analyses of the mean glucose levels during the intervention/MMTT, please refer to Fig 4.

There were no significant differences in mean blood glucose levels during any of the interventions, even though IW3 was borderline significantly lower compared to CON (Fig 4A). Nor were there any differences in glucose levels after the intervention (at time t = 60, 75 and 90 min) between interventions. In the end of the intervention (t = 60 min) mean incremental blood glucose levels were significantly lower after IW3 compared to CON (P < 0.05), whereas no significant differences were seen between IW1 and CON or between IW1 and IW3 (P > 0.05 for both).

Mean blood glucose levels during the MMTT (Fig 4B) were lower after IW3 compared to CON (10.3±3.0 vs. 11.1±3.3; P < 0.05), while IW1 (10.5±2.8) did not differ significantly from CON or IW3 (P > 0.05 for both). When only considering the last 2 or 3 hours of the MMTT, IW1 tended to be lower than CON (P = 0.06 and P = 0.08, respectively), with IW3 still being significantly lower than CON (P<0.05 for both). Moreover, glucose levels were significantly lower during both IW3 and IW1 compared to CON at several specific time points during the MMTT (Fig 3A). No significant difference in mean blood glucose levels were seen between IW1 and IW3 for any part of the MMTT (P > 0.05).

No significant differences in maximal glucose levels during the MMTT were seen between any of the interventions (Fig 4C). Nor were there any significant differences in mean- or maximal incremental glucose levels during the MMTT.

### Insulin levels and insulin sensitivity indices

No differences in baseline plasma insulin levels were seen between intervention days (P > 0.05 for all) (Fig 3B). Immediately after the intervention, insulin levels were lower for IW1 and IW3 compared to CON (P < 0.001 for both), whereas no difference was seen between IW1 and IW3 (P > 0.05). Fifteen minutes after termination of the intervention, insulin levels were higher after IW1 compared to CON (P < 0.001) and IW3 tended to be higher compared to CON (P < 0.1), whereas no difference was seen between IW1 and IW3 (P > 0.05). There were no significant differences in mean insulin levels during the MMTT between any of the trials (P > 0.05 for all comparisons), nor were there any significant differences in any of the measured insulin sensitivity indices between the trials (data not shown, P > 0.05 for all comparisons).

### RPE and preferred program

Data of RPE are shown in Table 2. No differences in RPE were seen between the exercise interventions during slow or fast intervals or when looking at mean. There was also no difference in overall RPE for the total intervention.

Eight of the eleven subjects preferred the IW3 intervention, whereas three preferred the IW1 intervention. Furthermore, eight subjects found the IW3 intervention to be the hardest one, while two subjects found the IW1 intervention to be the hardest one. One subject found the two exercise interventions equally hard. Five of the eight subjects that preferred IW3 also found this to be the harder intervention, whereas the three subjects that preferred IW1 all found IW3 hardest.

## Discussion

In this study we examined the effects of acute bouts of interval walking on postprandial glycemic control in type 2 diabetic patients. We found that one interval walking bout consisting of 3 min intervals improved postprandial blood glucose levels on a standardized meal initiated 30 min after completion of the exercise bout in subjects with type 2 diabetes as compared to no walking, which is consistent with our previous findings [6]. Whereas the mean postprandial glucose levels after the IW1 intervention did not differ significantly from the control intervention, the postprandial glucose levels after both IW3 and IW1 were significantly lower at specific time points throughout the MMTT compared to CON. Conversely, we found no differences in postprandial glucose levels between IW1 and IW3, neither for the MMTT overall or at any specific time points throughout the MMTT. Thus, tripling the number of intervals while keeping the walking speed, time spent on fast and slow walking and overall oxygen consumption constant did not further improve the glycemic control, suggesting that the cyclic pattern *per se* is not responsible for the beneficial effects of interval walking.

The two exercise sessions, IW1 and IW3, were matched regarding walking speed during slow and fast intervals, respectively, and total time spent on fast and slow walking. We observed that the mean oxygen consumption rate during exercise was the same in IW1 and IW3, suggesting that the walking economy did not depend on the length of the intervals and was apparently not affected by the number of accelerations and decelerations. These results made it possible to compare the two interval programs not only with regards to time and walking speed, but also with regards to energy expenditure and mean intensity.

Despite the fact that no difference in mean oxygen consumption between trials was seen, IW3 resulted in higher peak oxygen consumption values and lower nadirs during fast and slow intervals, respectively. Also, HR reached higher values during fast intervals and tended to reach lower values during slow intervals, when comparing IW3 to IW1, whereas lactate levels during exercise at all times were higher during IW3 compared to IW1. In this way, the physiological stress of exercise was probably higher during IW3 compared to IW1, something which has previously been found [18]. However, although we observed differences in lactate levels between the exercise interventions, we find it important to emphasize that the lactate levels were stable throughout the exercise interventions and at all time consistent with aerobic exercise [29]. Thus, we believe that the exercise sessions employed had lower peak intensities compared to previous studies [17,18], and, although no formal definition is available, we do not consider our exercise sessions to be ‘high-intensity interval exercise’.

Although far from significant, one might speculate that IW3 might improve postprandial glycemic control more than IW1, at least towards the end of the MMTT (Fig 3A). Also pointing in that direction, overall mean glucose levels during the MMTT differed significantly between IW3 and CON whereas no overall difference was found between IW1 and CON. Thus, the fact that IW3 reached higher peak intensity during the fast intervals compared to IW1, might indicate, that the peak exercise intensity is the most important factor.

However, since IW1 and IW3 differed both with regards to the peak intensity and with regards to the number of cycles, we cannot completely rule out that the more intervals and thereby the more accelerations and decelerations results in greater beneficial effect on blood glucose levels. If assuming that the peak exercise intensity also contributes to the beneficial effects seen, the effects of more cycles might be masked. Consequently, the question of whether peak exercise intensity is the more important factor for postprandial glycemic control in interval type exercise needs to be further investigated in future studies.

Although the insulin sensitivity indices we used in this study are not the golden standard for evaluating insulin sensitivity, there were no indications of any differences in insulin sensitivity between the trials. Even though the calculations were based on an MMTT instead of an OGTT as originally described and validated, which therefore limits the comparability to previous studies, our results indicate that the differences found in glycemic control between IW3 and CON, should be sought in insulin sensitivity-independent mechanisms.

For an exercise intervention to be successfully implemented in free-living settings, motivation and perceived exertion are both very important factors. The fact that eight out of eleven subjects preferred the IW3 program, despite that eight of the eleven found this program the hardest one, was paradoxical and surprising. Individual preferences and motivation should be considered when recommending an exercise program in order to enhance compliance. Moreover, while free-living adherence to our two types of interval exercise sessions is unknown, it is very important to assess since it has been suggested that the adherence to interval training regimes may be reduced when training is performed in a free-living setting [30].

The lack of difference in mean incremental glucose levels and maximal glucose levels between IW3 and CON during the MMTT is contrary to our previous findings, despite fairly similar subject characteristics in the two studies [6]. This may have been caused by the fact that the intensities during the interval exercise sessions fell short of the aimed intensities. This may also potentially explain the missing significant differences in overall mean absolute glucose values during the MMTT between IW1 and CON, where higher intensities might have led to significant beneficial effects. Furthermore, when looking at Fig 3A, one may suspect that the missing significant beneficial effect in mean glucose levels during the MMTT after IW1 compared to CON is due to low power. In this context, it should be noted that the sample size calculation performed was only based on comparisons between IW3 and CON. Thus, no formal sample size calculations were performed to assess differences between neither IW1 and CON, or IW3 and IW1. Moreover, since we had to exclude one subject from the analyses due to fluctuating estradiol levels, the achieved power for our primary outcome was 0.76 instead of the a priori chosen power of 0.80. Overall, we were probably underpowered to detect differences between IW1 and CON and largely underpowered to detect differences (or similarities) between IW3 and IW1 and, as such, no final conclusions regarding these comparisons can be drawn.

Some studies suggest that the effects of an acute bout of exercise on postprandial glycemic control in subjects with type 2 diabetes is more pronounced after the second meal [16,31]. These studies have however only investigated exercise with constant intensities, and whether the same is the case following acute interval-type exercise needs to be investigated in future studies. However, Gillen *et al*. found that postprandial glucose levels in subject with type 2 diabetes were lower for the subsequent 24 hours following a single interval-type exercise session compared to the control situation [32] and the same group [33] described that a similar interval-type exercise session improved glycemic control following breakfast on the following day compared to no exercise and continuous exercise in obese/overweight subjects. If glycemic control in our study was assessed not immediately after the exercise session, but instead throughout the day and the following day, one might speculate that the effects of interval exercise would be more pronounced and if IW1 would also have reached significant difference.

In conclusion, the current study showed that an acute bout of IW3 improved postprandial glycemic control on a standardized meal initiated 30 min after completion of the intervention compared to no walking. IW1 did not result in significant improvements in overall mean postprandial glucose levels compared to CON, but blood glucose levels at specific time points during the MMTT were lower in IW1 compared to CON. No differences in postprandial glycemic control were seen between IW1 and IW3. Thus, interval training with three minute intervals can be recommended for improving postprandial glycemic control in subjects with type 2 diabetes, whereas the importance of interval length needs to be further addressed in future studies.

## Supporting Information

S1 Protocol Study Protocol. (DOC)

S1 TREND Checklist (PDF)
