# Supplementary material for: The Acute Effects of Interval-Type Exercise on Glycemic Control in Type 2 Diabetes Subjects: Importance of Interval Length. A Controlled, Counterbalanced, Crossover Study
Source: PLoS One. 2016 Oct 3;11(10):e0163562. doi: 10.1371/journal.pone.0163562 (PMC5047444; doi:10.1371/journal.pone.0163562)
Supplement: S1 Protocol — (DOC) [file pone.0163562.s001.doc]

Study protocol

TITLE

The features behind the beneficial effects of Interval-walking

BACKGROUND

Physical activity is a first line treatment in type 2 diabetes, and the effect of physical activity on glycemic control is well-documented (1;2) but the optimal training approach is yet to be defined.

We have tested Interval-walking (IW) as a novel type of exercise and found that both a long-term training intervention (3) and a single exercise bout (Karstoft et al, JCEM, 2014) is superior to energy-expenditure and time-duration matched continuous walking (CW) exercise. IW, as it has been tested so far, consists of cycles of 3 minutes of fast walking and 3 minutes of slow walking.

The reason why IW is superior to CW upon improving glycemic control is unclear. In that context, at least two factors separate IW from CW: Peak exercise intensity and the alternating intensity pattern. Since some studies have found that continuous training with higher intensity results in greater improvements in metabolic variables compared to continuous training with lower intensity (4-7), the peak exercise intensity in IW may be responsible for the larger improvements in IW compared to CW. Conversely, other studies have found no differences between higher vs. lower continuous intensity training programs on metabolic variables (8-11), and one study has even found that a single lower intensity exercise session improves glycemic control compared with a single exercise session with higher intensity (12). Since most interval-type interventions have shown beneficial changes in metabolic variables (13-16), also when compared to energy-expenditure matched continuous-type interventions (17-20), it may be hypothesized that the alternating rather than the peak intensity is the important part of interval-based training.

With our recent evidence that IW improves glycemic control more than CW, it is of utmost importance to define which characteristics are responsible for the beneficial improvements. Therefore, the objective of this study is to assess the glycemic control in a standardized setting after the following interventions:

- An acute bout of ‘classical’ interval-walking (cycles of 3 min fast and 3 min slow walking; IW-3)
- An acute bout of ‘fast alternating’ interval-walking (cycles of 1 min fast and 1 min slow walking; IW-1)
- No walking (control; CON)

The exercise interventions will be matched with regards walking speed and time-duration.

It is hypothesized that the exercise bouts will improve glycemic control compared with CON but that no differential effect of the exercise bouts on glycemic control will be seen.

EXPERIMENTAL DESIGN

*Sample Size:* Based on our previous study examining glycemic control after acute IW(-3) compared to CON in type 2 diabetic subjects (standardized effect size = 0.95, (21)), power calculations were performed using G*Power (v3.1.9.2, Düsseldorf, Germany). To account for multiplicity, α was set at 0.017 (0.05 / 3), and 1-β was set at 0.80. These analyses indicated that at least 12 subjects should complete the study. If a subject does not complete the study, he or she will be replaced by another subject, but the total number of subjects included will not exceed 24.

*Recruitment, information and informed consent:* Potential subjects are recruited through direct contact, advertising on "www.forsøgsperson.dk" and advertising in local newspapers. Potential subjects contact one of the project participants by telephone and are orally informed about the study. If no exclusion criteria are identified in the phone, an information meeting date is arranged, and the potential subject is informed about the possibility of inviting a private counselor to the information meeting. At least three days prior to the information meeting, written project information including consent form will be sent to the potential subject and he/she will be encouraged to read the information thoroughly.
At the information day, extensive oral information about the scientific study will be given by one of the scientific participants. The information will be given in a closed room where only the scientific participant, the potential subject and the potential private counselor will be present. After the oral information has been given, the potential subject will be asked if he/she wants time to consider before he/she is ready to decide whether or not to participate in the study. If the potential subject does not want time to consider potential participation but is ready to decide and wants to participate in the study, the informed consent form will be signed and the screening will commence. If the potential subject wants additional time to consider whether or not to participate in the study, an agreement about a telephone meeting after approx. one week is made. The subject will be informed of the possibility of contacting one of the scientific participants by telephone, in case of questions. If the potential subject wants to participate in the study, a screening will be arranged and the signed consent form will be brought to the screening by the subject.

*Subjects and screening:* A total of n=12 type 2 diabetes patients will initially be included in the study. A medical examination, medical history, blood chemistry screen, ECG, oral glucose tolerance test (OGTT), Dual x-ray absorptiometry (DXA) scan and a physical fitness test (VO_2_max test) will be performed. If an individual meets the inclusion criteria, informed oral and written consent will be obtained prior to participation in the study, in accordance with guidelines developed by the ethical committees.

*Inclusion Criteria:*

- Type 2 diabetes mellitus
- Age >30 y
- BMI >18 but <40 kg/m^2^
- Agreement to pause non-insulin anti-diabetic medication from 2 days before to the end of each intervention

*Exclusion Criteria:*

- Pregnancy
- Smoking
- Contraindication to increased levels of physical activity (22)
- Insulin dependence
- Evidence of thyroid, liver, lung, heart or kidney disease

STUDY DESIGN

Subjects will be instructed to ingest the same food at each day preceding the interventions, to avoid alcohol and caffeine for 24 hours prior to the interventions and to abstain from vigorous physical activity for 48 hours prior to the interventions.

*Interventions:* The 3 interventions will be performed in counter-balanced order and separated by at least one week.

Each intervention day will, besides the differences in interventions, be identical and consist of the following:

- t = -30 min: Arriving fasting (≥8 hours) at lab, acclimatization
- t = 0 min: Blood sample, initiation of intervention (IW-3, IW-1, CON)
- t = 60 min: Blood sample, termination of intervention
- t = 90 min: Initiation of Mixed Meal Tolerance Test (MMTT)
- t = 270 min: Termination of MMTT

METHODS

*Interventions:* At the screening day, a graded walking VO_2_peak-test (23-25) will be performed using a portable indirect calorimetry system. Based on the peak VO_2_-rate obtained, two individualized exercise bouts (interventions) will be designed (an interval-walking bout and a continuous-walking bout). Based on the findings from our previous study, the intensities of ‘classical’ and ‘fast alternating’ interval- (fast and slow) walking will be 89 and 54% of the peak VO_2_-rate, respectively (3). To ensure comparable energy-expenditure, IW-3 and IW-1 bouts will be of 60 min’s duration. All exercise bouts will be performed at a treadmill (Technogym Runrace, Gambettola, Italy), using continuous indirect calorimetric measurements (CPET, Cosmed, Italy) and heart rate monitoring (Polar Electro, Holte, DK). Rate of perceived exertion will be assessed during and after the exercise interventions (26). For the control intervention, subjects will be placed supine for one hour.

*MMTT:* A standardized liquid MMTT (300 kcal with a macronutrient composition that resembles a normal meal; 55% carbohydrates, 30% fat and 15% protein) will be performed after each intervention.

30 minutes after conclusion of the intervention, the MMTT will commence. Blood sampling for subsequent measurements of glucose (NaF plasma tubes), insulin and C-peptide (serum tubes) and incretin and glucagon hormones (EDTA plasma tubes containing aprotinin (500 kiU/mL) and a dipeptidyl peptidase 4 inhibitor (0.01 mmol/L diprotin A) will be performed at the following time points: -30, 0, 30, 60, 90 (start of MMTT), 105, 120, 135, 150, 180, 210, 240 and 270 min. Blood samples for plasma collection will immediately be placed on ice and subsequently centrifuged (2000 g, 15 minutes, 4°C). Samples for serum collection will be left at room temperature for 30 min and centrifuged. Samples will be stored at -80°C until analysis. Plasma glucose will be determined by an enzymatic colorimetric assay (P-Modular, Roche, Switzerland), serum insulin and c-peptide will be measured by electrochemiluminescence immunoassay (E-Modular, Roche, Switzerland) and incretins and glucagon will be measured by radioimmunoassay assays.

RISKS, ADVERSE EFFECTS AND DISCOMFORT

*VO_2_-max test:* A physical fitness test, where subjects must put in maximum effort. This will cause some degree of breathlessness. VO_2_max test is a standard method used for scientific purposes in our laboratory.
*DXA scan:* Is not expected to cause significant discomfort. The radiation acquired is so small that it doesn’t cause any risk to subjects. DXA-scan is a standard method used for scientific purposes in our laboratory.

*Pausation of non-insulin anti-diabetic medication:* In order to evaluate the effect of the interventions, it is necessary for subjects to pause medication the days prior to and on the intervention days. It is considered to be without any risk to pause these drugs for shorter time periods.
*Blood sampling:* Will cause minor discomfort in terms of a venous catheter. There is theoretically a risk for infections introduced through the catheter. The blood volume collected is so small that it will cause no symptoms. Blood sampling is a standard method used for scientific purposes in our laboratory.

SUBJECT’S PHYSICAL AND MENTAL INTEGRITY AND PRIVACY

The study will be reported to “Datatilsynet” through Rigshospitalets joint review.

The “Lov om behandling af personoplysninger” will be respected.

ECONOMICAL SUPPORT

Kristian Karstoft has initiated the study. Different private foundations will be applied for economical support (see attached budget). If economical support is obtained, the ethical committee will be notified. Centre for Physical Activity Research (CFAS) will cover the costs not covered by external foundations. No commercial institutions have been or will be applied for economical support. None of the scientific participants have any disclosures to declare.

Subjects will be paid a fee of DKK 2,000 for participation in the study. The fee is taxable. The fee covers discomfort, time consumption and travel expenses. The fee is paid by the end of the subject's participation. If the subject is excluded or choose to withdraw from the trial, a fee equal to the part of the study the subject has completed will be paid.

ETHICAL ASPECTS

The project is, as described above, expected to result in limited risks, adverse effects and discomfort to the subjects. The subjects will benefit from the study in terms of a thorough medical examination and in terms of introduction to effective training methods.
For research in general, the study is sound and important, and it will contribute to our knowledge about how to increase public health in a large and growing cohort of people with lifestyle diseases.

STATISTICAL ANALYSES

All data will be tested for normality and homogeneity of variance using Kolmogorov-Smirnov and Levene tests. Variables that diverge from parametric assumptions will be log-transformed prior to analysis. Variables of interest will be compared via one-way repeated-measure analysis of variance (RM-ANOVA), and where significant interactions arise, Bonferroni post hoc tests will be applied to identify specific differences between means. Relationships between changes in variables will be examined using regression analyses.

BIOLOGICAL MATERIAL

Blood will be collected in the study. This will be used to assess the potential differential changes in variables following the interventions.
Approx. 100 mL blood will be collected per trial day. Total blood sampling will be approx 400 mL. The biological material will, during the study, be stored in a research-biobank. After the study is completed (August 31^st^, 2016), the biological material will be transferred to a biobank for a maximum of 20 years and after that, it will be destroyed. If any later studies want to make use of the biological material, this will only take place following approval by the ethical committee.
The biological material will not leave Denmark.

EXPECTED OUTCOMES

It is expected that this project will show that the interval-walking exercise bouts are superior to no walking upon improvements in glycemic control in type 2 diabetes patients. This will further improve our understanding of exercise as a concept for fighting lifestyle diseases and potentially lead to changes in the way future exercise interventions are designed and implemented in patient care.

DISSEMINATION

Data will be presented at the American Congress of Sports Medicine (ACSM’s annual meeting) in 2015. At least one manuscript will be produced from the data and published in an international peer-reviewed journal. Positive, negative and inconclusive results will be published.

STUDY LOCATION

Centre for Physical Activity Research (CFAS)
Rigshospitalet
Tagensvej 20, section M7641
DK-2100 Copenhagen
Denmark

SCIENTIFIC PARTICIPANTS

Kristian Karstoft, MD, responsible investigator, Post doc, Centre for Physical Activity Research (CFAS), Rigshospitalet

Thomas PJ Solomon, cand. scient, Ph.D., associate professor., Department of Biomedical Sciences, Panum, University of Copenhagen,

Research assistent (un-named), Centre for Physical Activity Research (CFAS), Rigshospitalet

Bente Klarlund Pedersen, MD, DMSc, professor, Centre for Physical Activity Research (CFAS), Rigshospitalet

Reference List

1. Snowling NJ, Hopkins WG (2006) Effects of different modes of exercise training on glucose control and risk factors for complications in type 2 diabetic patients: a meta-analysis. Diabetes Care 29: 2518-2527

2. Boule NG, Haddad E, Kenny GP, Wells GA, Sigal RJ (2001) Effects of exercise on glycemic control and body mass in type 2 diabetes mellitus: a meta-analysis of controlled clinical trials. JAMA 286: 1218-1227

3. Karstoft K, Winding K, Knudsen SH, et al (2012) The Effects of Free-Living Interval-Walking Training on Glycemic Control, Body Composition, and Physical Fitness in Type 2 Diabetes Patients. Diabetes Care

4. Irving BA, Davis CK, Brock DW, et al (2008) Effect of exercise training intensity on abdominal visceral fat and body composition. Med.Sci.Sports Exerc. 40: 1863-1872

5. DiPietro L, Dziura J, Yeckel CW, Neufer PD (2006) Exercise and improved insulin sensitivity in older women: evidence of the enduring benefits of higher intensity training. J.Appl.Physiol 100: 142-149

6. Balducci S, Zanuso S, Nicolucci A, et al (2010) Anti-inflammatory effect of exercise training in subjects with type 2 diabetes and the metabolic syndrome is dependent on exercise modalities and independent of weight loss. Nutr.Metab Cardiovasc.Dis. 20: 608-617

7. Boule NG, Kenny GP, Haddad E, Wells GA, Sigal RJ (2003) Meta-analysis of the effect of structured exercise training on cardiorespiratory fitness in Type 2 diabetes mellitus. Diabetologia 46: 1071-1081

8. Hansen D, Dendale P, Jonkers RA, et al (2009) Continuous low- to moderate-intensity exercise training is as effective as moderate- to high-intensity exercise training at lowering blood HbA(1c) in obese type 2 diabetes patients. Diabetologia 52: 1789-1797

9. O'Donovan G, Kearney EM, Nevill AM, Woolf-May K, Bird SR (2005) The effects of 24 weeks of moderate- or high-intensity exercise on insulin resistance. Eur.J.Appl.Physiol 95: 522-528

10. Houmard JA, Tanner CJ, Slentz CA, Duscha BD, McCartney JS, Kraus WE (2004) Effect of the volume and intensity of exercise training on insulin sensitivity. J.Appl.Physiol 96: 101-106

11. Braun B, Zimmermann MB, Kretchmer N (1995) Effects of exercise intensity on insulin sensitivity in women with non-insulin-dependent diabetes mellitus. J.Appl.Physiol (1985.) 78: 300-306

12. Manders RJ, Van Dijk JW, van Loon LJ (2010) Low-intensity exercise reduces the prevalence of hyperglycemia in type 2 diabetes. Med.Sci.Sports Exerc. 42: 219-225

13. Richards JC, Johnson TK, Kuzma JN, et al (2010) Short-term sprint interval training increases insulin sensitivity in healthy adults but does not affect the thermogenic response to beta-adrenergic stimulation. J.Physiol 588: 2961-2972

14. Little JP, Gillen JB, Percival M, et al (2011) Low-volume high-intensity interval training reduces hyperglycemia and increases muscle mitochondrial capacity in patients with type 2 diabetes. J.Appl.Physiol

15. Mourier A, Gautier JF, De KE, et al (1997) Mobilization of visceral adipose tissue related to the improvement in insulin sensitivity in response to physical training in NIDDM. Effects of branched-chain amino acid supplements. Diabetes Care 20: 385-391

16. Babraj JA, Vollaard NB, Keast C, Guppy FM, Cottrell G, Timmons JA (2009) Extremely short duration high intensity interval training substantially improves insulin action in young healthy males. BMC.Endocr.Disord. 9: 3

17. Tjonna AE, Lee SJ, Rognmo O, et al (2008) Aerobic interval training versus continuous moderate exercise as a treatment for the metabolic syndrome: a pilot study. Circulation 118: 346-354

18. Karstoft K, Winding K, Knudsen SH, et al (2013) The Effects of Free-Living Interval-Walking Training on Glycemic Control, Body Composition, and Physical Fitness in Type 2 Diabetes Patients. Diabetes Care 36: 228-236

19. Daussin FN, Zoll J, Dufour SP, et al (2008) Effect of interval versus continuous training on cardiorespiratory and mitochondrial functions: relationship to aerobic performance improvements in sedentary subjects. Am.J.Physiol Regul.Integr.Comp Physiol 295: R264-R272

20. Mitranun W, Deerochanawong C, Tanaka H, Suksom D (2013) Continuous vs interval training on glycemic control and macro- and microvascular reactivity in type 2 diabetic patients. Scand.J.Med.Sci.Sports

21. Karstoft K, Christensen CS, Pedersen BK, Solomon TP (2014) The acute effects of interval- vs. continuous-walking exercise on glycemic control in subjects with type 2 diabetes: a cross-over, controlled study. J.Clin.Endocrinol.Metab jc20141837

22. Pedersen BK, Saltin B (2006) Evidence for prescribing exercise as therapy in chronic disease. Scand.J.Med.Sci.Sports 16 Suppl 1: 3-63

23. Nemoto K, Gen-no H, Masuki S, Okazaki K, Nose H (2007) Effects of high-intensity interval walking training on physical fitness and blood pressure in middle-aged and older people. Mayo Clin.Proc. 82: 803-811

24. Nose H, Morikawa M, Yamazaki T, et al (2009) Beyond epidemiology: field studies and the physiology laboratory as the whole world. J.Physiol 587: 5569-5575

25. Morikawa M, Okazaki K, Masuki S, et al (2011) Physical fitness and indices of lifestyle-related diseases before and after interval walking training in middle-aged and older males and females. Br.J.Sports Med. 45: 216-224

26. Borg GA (1982) Psychophysical bases of perceived exertion. Med.Sci.Sports Exerc. 14: 377-381
